# Supplementary material for: Clinical characterization of a cohort of patients treated for systemic lupus erythematosus in Colombia: A retrospective study
Source: PLoS One. 2023 May 18;18(5):e0285889. doi: 10.1371/journal.pone.0285889 (PMC10194955; doi:10.1371/journal.pone.0285889)
Supplement: Attachment — Submitted filename: Response to reviewers LES (10-08-2022).docx [file pone.0285889.s001.docx]

PONE-D-22-14695R1
Clinical characterization of a cohort of patients under treatment for systemic lupus erythematosus in Colombia: a retrospective study

Dear Edrian Nim Tolentino
PLOS ONE

We have reviewed all your concerns and we respond to each of them.

1. Thank you for stating the following in the Competing Interests section:
This study received funding from GSK. The funder had the following involvement with the study: GSK did not participate in the management or data analysis of the study information.   CDR and AGR are GSK employees; JMA, AGG and MMD have developed studies funded by GSK, Pfizer, Biotoscana, Sanofi, Sanofi-Pasteur, Abbot, Tecnoquímicas, Bayer, Novartis.

**Response/ Thank you.**

Please confirm that this does not alter your adherence to all PLOS ONE policies on sharing data and materials, by including the following statement: "This does not alter our adherence to  PLOS ONE policies on sharing data and materials.” (as detailed online in our guide for authors <http://journals.plos.org/plosone/s/competing-interests>).  If there are restrictions on sharing of data and/or materials, please state these. Please note that we cannot proceed with consideration of your article until this information has been declared.

**Response/ confirmed, we add “This does not alter our adherence to  PLOS ONE policies on sharing data and materials”**

Please include your updated Competing Interests statement in your cover letter; we will change the online submission form on your behalf.

**Response/ confirmed, we add “This does not alter our adherence to  PLOS ONE policies on sharing data and materials” in cover letter.**

2. Please provide additional details regarding participant consent. In the Methods section, please ensure that you have specified (1) whether consent was informed and (2) what type you obtained (for instance, written or verbal). If your study included minors, state whether you obtained consent from parents or guardians. If the need for consent was waived by the ethics committee, please include this information.

**Response/ we add in Ethics statement**

**According to Colombian regulations (Decree 8430 of 1993), risk-free research that takes information from clinical records does not require informed consent. The research was endorsed by the Bioethics Committee of the Universidad Tecnológica de Pereira in the risk-free category (Approval code: CBE 132018). The principles established by the Declaration of Helsinki were respected. No personal data from any of the research participants were considered.**

3. We note your current Data Availability statement is:

"Yes - all data are fully available without restriction"
"This text is appropriate if the data are owned by a third party and authors do not have permission to share the data."

**Response/ ok, we will make the correction in the journal application.**

We also note you included the following, additional information in your response to reviewers:

"The original contributions presented in the study are included in the article/supplementary material, further inquiries can be directed to the corresponding author/s." The name of the information repository is: [protocols.io](http://protocols.io/)
Code availability: [dx.doi.org/10.17504/protocols.io.bzufp6tn](http://dx.doi.org/10.17504/protocols.io.bzufp6tn)”

PLOS journals require authors to make the minimal data set publicly available without restriction at the time of publication. PLOS defines the “minimal data set” as consisting of the data set used to reach the conclusions drawn in the manuscript with related metadata and methods, and any additional data required to replicate the reported study findings in their entirety. This includes:

- The values behind the means, standard deviations and other measures reported;
- The values used to build graphs;
- The points extracted from images for analysis.

Can you please address the following and clarify where the relevant data are located:

a.) Can you please clarify whether the minimal dataset for this study are uploaded to [protocols.io](http://protocols.io/) and available via the provided DOI ([dx.doi.org/10.17504/protocols.io.bzufp6tn](http://dx.doi.org/10.17504/protocols.io.bzufp6tn))?

**Response/ Yes, the minimal dataset for this study are uploaded to protocols.io and available via the provided DOI (dx.doi.org/10.17504/protocols.io.bzufp6tn)**

b.) If not, are the data third party data (i.e., data not owned or collected by the author(s))? If these are indeed third party data, please explain how others can access these datasets and confirm that others would be able to access these data in the same manner as the authors. Please also confirm that the authors did not have any special access privileges that others would not have.

**Response/ All data is owned by the authors. In addition, the data was correctly anonymized. as it says in methods, where it is indicated " The principles established by the Declaration of Helsinki were respected. No personal data from any of the research participants were considered”.**

c.) If not, but there are ethical or legal restrictions on sharing a de-identified data set, please explain them in detail (e.g., data contain potentially identifying or sensitive information) and who has imposed them (e.g., a governmental body, an ethics committee, etc.). Please also provide non-author contact information* for a data access committee, ethics committee, or other institutional body to which data requests may be sent.

**Response/ N/A**

* PLOS’ Data Policy states that authors cannot be the sole named individuals responsible for ensuring data access (<https://journals.plos.org/plosone/s/data-availability#loc-unacceptable-data-access-restrictions>).

**Response/ The anonymized data are public since the manuscript was written and are available at the corresponding link for anyone who wants to consult them.**

We will update your Data Availability statement on your behalf using the information you provide.

The authors
