# Supplementary material for: Clinical characterization of a cohort of patients treated for systemic lupus erythematosus in Colombia: A retrospective study
Source: PLoS One. 2023 May 18;18(5):e0285889. doi: 10.1371/journal.pone.0285889 (PMC10194955; doi:10.1371/journal.pone.0285889)
Supplement: Attachment — Submitted filename: comment for PLOS.docx [file pone.0285889.s002.docx]

Overall the paper is well revised; I just have few comments

On Line 39 and line 101 please re-write again, don’t put full stops before finishing a sentences, re-write it again.

Why didn’t you give any recommendation for the missing variables (or incomplete) like emergency department visits, costs associated with hospital care, ambulatory health records

Do you think you have measured economic burden well? Can you say something about that on the paper?

On the **data source** can you describe the time frame of your data collection.
