# Supplementary material for: Clinical characterization of a cohort of patients treated for systemic lupus erythematosus in Colombia: A retrospective study
Source: PLoS One. 2023 May 18;18(5):e0285889. doi: 10.1371/journal.pone.0285889 (PMC10194955; doi:10.1371/journal.pone.0285889)
Supplement: Attachment — Submitted filename: Response to Reviewers 18-10-22.docx [file pone.0285889.s003.docx]

**Response to reviewers**

**Dear Editor**

**Journal:** PLOS ONE

**Manuscript ID number:** PONE-D-22-14695R1

**Title:** Clinical characterization of a cohort of patients treated for systemic lupus erythematosus in Colombia: a retrospective study

We have reviewed the manuscript and responded to each concern. In addition, AJE, who performed the translation, was asked to review the coherence and readability.

**Reviewer #1:** Overall the paper is well revised; I just have few comments

On Line 39 and line 101 please re-write again, don’t put full stops before finishing a sentences, re-write it again.

**Response: We corrected the sentences on lines 39 and 101 regarding the study design.**

Why didn’t you give any recommendation for the missing variables (or incomplete) like emergency department visits, costs associated with hospital care, ambulatory health records

**Response: In the conclusions section, we added a sentence recommending future studies that include these variables. Ambulatory health records were the main source of information in the study.**

Do you think you have measured economic burden well? Can you say something about that on the paper?

**Response: We think that economic burden was adequately measured using the proposed methodology. The limitations regarding cost calculations were addressed. We think it is unnecessary to further discuss this issue in the paper.**

On the data source can you describe the time frame of your data collection.

**Response: The data frame is described in the subsection “Study design and settings” and corresponded to July 1, 2016, through June 30, 2017.**

**Reviewer #2:** Minor Comment

-English editing is required. Coherence and readability of the manuscript need an improvement.

**Response: The final version of the manuscript was edited by American Journal Experts. We hope this editing addresses this comment. We add the certificate.**

**The authors**
