# Supplementary material for: Clinical characterization of a cohort of patients treated for systemic lupus erythematosus in Colombia: A retrospective study
Source: PLoS One. 2023 May 18;18(5):e0285889. doi: 10.1371/journal.pone.0285889 (PMC10194955; doi:10.1371/journal.pone.0285889)
Supplement: Attachment — Submitted filename: Plos review.docx [file pone.0285889.s004.docx]

Review for : Clinical characterization of a cohort of patients treated for systemic lupus erythematosus in Colombia: a retrospective study

The authors have addressed all comments given last time and has revised the manuscript.

Just minor comments.

**Abstract**

I wouldn’t advise use of abbreviation on the abstract section of manuscript

**Methods**

Does the outcome of SLE not different according to age? How did you take account of that during your analysis? Since you included all age? But on the result it says 18-86..so you should either say we included all ages that are 18 and above

Your sample size is totally not clear? It needs reference, show the formula you used, what does expect frequency 50% mean??? For retrospective cohort study there is a formula ..

On the ethical declaration section good to include reference for Colombian regulations (Decree 8430 of 1993)

**Result**

Either report the age result in mean and SD or with the category mentioning both at the same time is not necessary.

What I understood is this is observation retrospective study or retrospective cohort study do please report the incidence of SLE don’t talk about prevalence

**Limitation**

How does excluding incomplete data such as clinometric cases affected or limited your study in any way?

How many cards did you initially find? And how many of them were incomplete? Mention this in the result first section I think this information is relevant to mention as limitation too…you said some variables are incomplete so what is that implication on your finding.. what were the variables
