# Supplementary material for: Clinical characterization of a cohort of patients treated for systemic lupus erythematosus in Colombia: A retrospective study
Source: PLoS One. 2023 May 18;18(5):e0285889. doi: 10.1371/journal.pone.0285889 (PMC10194955; doi:10.1371/journal.pone.0285889)
Supplement: Attachment — Submitted filename: Response to Reviewers 24-03-23.docx [file pone.0285889.s005.docx]

**Response to reviewers**

**Dear Editor**

**Journal:** PLOS ONE

**Manuscript ID number:** PONE-D-22-14695R2

**Title:** Clinical characterization of a cohort of patients treated for systemic lupus erythematosus in Colombia: a retrospective study

We have reviewed the manuscript and responded to each concern.

**Comments to the Author**

**Reviewer #1:**

The authors have addressed all comments given last time and has revised the manuscript.

Just minor comments.

**Abstract**

I wouldn’t advise use of abbreviation on the abstract section of manuscript

***R/ We have avoided the use of systemic lupus erythematosus (SLE) abbreviation. we can remove the acronym and leave the full words.***

**Methods**

Does the outcome of SLE not different according to age? How did you take account of that during your analysis?

***R/ We have now included the results of the SLEDAI in table 1 according to the age categories in order to show the differences according to age.***

Since you included all age? But on the result it says 18-86..so you should either say we included all ages that are 18 and above

***R/ Corrected, thank you***

Your sample size is totally not clear? It needs reference, show the formula you used, what does expect frequency 50% mean??? For retrospective cohort study there is a formula ..

***R/ This is an observational, retrospective study. We used data from a cohort of patients with SLE, but the study design was not an analytical cohort study (we did not have an exposed and unexposed group comparison). An expected frequency of 50% was used to reach the highest sample size. We have now included the formula and references (17 and 18). We changed the term “expect frequency” for “estimated proportion”.***

On the ethical declaration section good to include reference for Colombian regulations (Decree 8430 of 1993)

***R/ Included (reference 19)***

**Result**

Either report the age result in mean and SD or with the category mentioning both at the same time is not necessary.

***R/ We have eliminated the age groups from the text.***

What I understood is this is an observation retrospective study or retrospective cohort study do please report the incidence of SLE don’t talk about the prevalence

***R/ We did not consider the new cases of SLE during the time. We had a database of patients already diagnosed with SLE. Thus, the incidence of SLE can not be calculated. However, we did show the incidence of certain SLE manifestations during the follow-up year, such as lupus nephritis***.

**Limitation**

How does excluding incomplete data such as clinometric cases affected or limited your study in any way?

***R/ Only patients with an initial SLEDAI were included in the sample, and thus the results showed in the manuscript for the 413 patients include this clinimetric information. The limitation is for the final SLEDAI results, which were available in only 227 patients. This is already explicitly stated in the second paragraph of the results section and in the limitations (in the discussion section).***

How many cards did you initially find? And how many of them were incomplete? Mention this in the result’s first section I think this information is relevant to mention as a limitation too…you said some variables are incomplete so what is that implication on your finding.. what were the variables

***R/ We do not have the data of initial registries that were incomplete. For the variables included in the study, only the last SLEDAI and the exact date of diagnosis had missing values. This is now included in the limitations. When we say that some variables were not complete due to the origin of the data, we also intend to mention that some other variables were not available (data was retrospective). We have now added this in the limitations.***

**Reviewer #2**

I would like to appreciate the authors for taking their time to correctly address the issues raised during peer-review process. All the comments are satisfactorily addressed. This paper is a candidate for publication.

***R/ thank you***

**The authors**
